# Is the Piglet Grimace Scale (PGS) a Useful Welfare Indicator to Assess Pain after Cryptorchidectomy in Growing Pigs?

**DOI:** 10.3390/ani10030412

**Published:** 2020-03-02

**Authors:** Cecilia Vullo, Sara Barbieri, Giuseppe Catone, Jean-Marie Graïc, Marco Magaletti, Ambra Di Rosa, Ambra Motta, Carlo Tremolada, Elisabetta Canali, Emanuela Dalla Costa

**Affiliations:** 1 Dipartimento di ChiBioFarAm, Università degli Studi di Messina, 98122 Messina, Italy; cvullo@unime.it; 2 Dipartimento di Medicina Veterinaria, Università degli Studi di Milano, 20133 Milano, Italy; sara.barbieri@unimi.it (S.B.); ambra.motta@unimi.it (A.M.); carlo.tremolada@guest.unimi.it (C.T.); elisabetta.canali@unimi.it (E.C.); 3 Dipartimento di Scienze Veterinarie, Università degli Studi di Messina, 98168 Messina, Italy; gcatone@unime.it (G.C.); dirosaa@unime.it (A.D.R.); 4 Dipartimento di Biomedicina Comparata e Alimentazione, Università degli Studi di Padova, 35020 Padova, Italy; jeanmarie.graic@unipd.it; 5 Service de la Consommation et des affaires Vétérinaires de l’Etat de Genève (SCAV), 1211 Geneva, Switzerland; marco.magaletti@etat.ge.ch

**Keywords:** pig, piglet grimace scale, pain, castration, behavior, pain assessment

## Abstract

**Simple Summary:**

On farms, pigs undergo husbandry procedures that can be painful. However, pain assessment in pig farming faces practical limitations, pertaining to the lack of reliable and feasible tools. This work aimed to investigate whether the Piglet Grimace Scale (PGS), a facial-expression-based pain coding system, could be usefully applied to assess pain associated with on-farm surgery in growing pigs. Ten pigs affected by cryptorchidism (a congenital anomaly which requires surgical intervention) were assessed before and 6 h after surgery. The authors found that the PGS score increased after surgery, and therefore, that the PGS is a potentially effective method to assess pain associated with surgery in growing pigs.

**Abstract:**

Pig cryptorchidism is a congenital anomaly, which requires surgical intervention. Pain assessment in pig farming has some limitations and consumers are increasingly concerned about suffering linked to on-farm procedures. The PGS seems to be a promising tool for pain assessment in piglets, although it has not been investigated whether this tool is applicable to growing pigs. This study was designed to determine whether the PGS could be useful to assess pain in growing pigs undergoing on-farm cryptorchidectomy. Ten mixed-breed cryptorchid pigs were pre-medicated with azaperone and ketamine. Anesthesia was induced and maintained using IV sodium based. Pigs were filmed pre- and 6 h post-surgery to evaluate their behavior (scan sampling every minute). Besides, 36 pictures of the face expressions (18 pre- and 18 6 h post-surgery) were scored with the PGS by three treatment-blind observers. The pre-surgery pig’s activity ratio was 59%, while it was 2% 6 h post-surgery. While the PGS inter-observer reliability was excellent (Interclass Correlation Coefficient value of 0.87), the PGS score increased significantly in pigs 6 h post-surgery (Paired sample *t*-test, *p* = 0.02). The PGS proved to be a potentially effective method to assess pain associated with cryptorchidectomy. However, further validation studies are required to validate this tool for other potentially painful procedures.

## 1. Introduction

Cryptorchidism is a congenital anomaly in pigs in which testes are retained in the abdominal cavity after the normal time for testicular descent. Products of cryptorchid pigs are downgraded on the market due to their “boar taint” [1]. Boar taint is an offensive taste and smell, due to an androstenone concentration in fat [2]; which becomes evident when cooking and eating pork products. For this reason, on-farm surgical castration is usually suggested to remove the intra-abdominal testes.

Surgical castration is a painful procedure (for a review see [3]): scientific literature has proven that, within the first few hours after the procedure, concentrations of cortisol, adrenocorticotropic hormone (ACTH), and lactate are elevated in the plasma of castrated piglets compared to control (which are only handled) [4]. White and colleagues reported that in castrated piglets, heart rate, respiration rate, and blood pressure increased [5]. Behavioral changes are also reported to be linked with surgical castration: Piglets reduce nursing behavior and are more likely to spend time in social isolation [4,6]. For all these reasons, consumers are increasingly concerned about pig welfare and demand that the industry aims at reducing pain and suffering linked with this husbandry procedure. According to the legislation in force in the European Union (Council Directive 2008/120/EC of 18 December 2008) “if pig castration is practiced after the seventh day of life, it shall only be performed under anesthetic and additional prolonged analgesia by a veterinarian”. However, the main obstacle that farmers and veterinarians reported in using prolonged analgesia in pigs is the recognition and quantification of pain [7]. In pig farming, pain assessment is characterized by a low specificity and practical limitations (for a review see [8]), highlighting the lack of a reliable and usable tool [9]. For instance, physiological indicators such as cortisol level, heart rate or respiratory rate are not specific enough for pain assessment, and furthermore, they have been validated in experimental settings, but their application on-farm is difficult as it requires the use of a heart rate monitor and blood sample collection [8]. On the other hand, behavioral indicators are specific to painful conditions and are reliable, but the frequency of their expression can be low, with a high between-subject variation, resulting in an impractical time-consuming assessment on-farm [8].

Recently, grimace scales, facial expressions-based pain coding systems, have been developed for pain assessment in several species, including pigs (for a review see [10]). Grimace scales have several advantages: They are non-invasive as they require short observation of the animal, they are easily applicable and they seem promising to rapidly detect pain [10,11]. For all these reasons, they could be a suitable indicator to be applied in pig farms to detect pain, not only in piglets undergoing husbandry procedures [9,12], but also for older animals, such as growing pigs or saws. The Piglet Grimace Scale (PGS) [9,11,12] incorporates three Facial Action Units (FAUs): ear position, orbital tightening, and cheek tightening/nose bulge. Ear position and cheek tightening/nose bulge are scored on a 3-point scale (0–2), with zero indicating that the assessor is confident that the action unit is not present, one indicating that the action unit is moderately present, and two indicating that the assessor is confident that the action unit is obviously present. Orbital tightening is scored on a 2-point scale (0 = not present; 1 = present).

The PGS seems to be a promising tool of assessing pain in piglets [9,11,12]. Piglets are neonate animals, meaning that during their rapid growth, morphological changes in their body and in their facial integument and musculature will thoroughly affect their expression range. Interestingly, some of the FAUs in the PGS corresponded to those reported in other species (e.g., temporal tension, orbital tightening), where they are assessed on adult animals. To date, no studies have been carried out to investigate whether the PGS can be applied to growing pigs. For all the above-mentioned reasons, this study aimed to determine whether the FAUs included in the PGS can be useful to assess pain in growing pigs undergoing on-farm cryptorchidectomy.

## 2. Materials and Methods

This study is a veterinary observational study, all the animals involved underwent to routine veterinary surgery on-farm. The “Institutional Animal Ethics Committee on the care and use of experimental animals” approved the study design as veterinary study (protocol number E81AC.8/A). Ethical approval for behavioral observations was granted without a formal application because they are deemed not likely to cause pain, suffering, or distress.

### 2.1. Animals and Husbandry

Ten healthy mixed-breed (Large White x Duroc) male pigs (mean weight 35 ± 3.52 kg; mean age 73 ± 10.6 days) affected by unilateral abdominal cryptorchidism, were recruited and housed in an indoor commercial pig farm. Pigs were kept in 2 collective pens (5 pigs/pen) on a partially slatted floor, with a floor space of 1.50 m^2^ per pig. Each pen was equipped with a nipple drinker and water was constantly available. The pigs were fed ad libitum twice a day with pellets of a standard commercial diet (metabolizable energy: 9.7 MJ; crude protein: 18 g; lysine: 9.5 g). About 2 kg of straw was provided daily on the lying area of the pen (according to Council Directive 2008/120/EC). The building was naturally ventilated and artificial lighting was supplied to provide 8 h of light per day.

### 2.2. Anaesthetic Protocol and Surgical Procedure

Food was withheld for 12 h and water for 30 min prior to anesthesia. The pigs were pre-medicated with a combination of azaperone (2 mg/kg; Stresnil^®^, Elanco Italia S.p.A., Sesto Fiorentino, Italy) and ketamine (10 mg/kg; Ketavet 100^®^, Intervet, Aprilia, LT, Italy), administered by intramuscular injection into the neck behind the base of the ear by using a 19 gauge needle connected to a line extension (75 cm, 1.3 mL volume, Sidam Medical Device^®^, Italy). Pain on injection was scored using a simple descriptive scale modified [13]: score 0 = no pain (the animal is quiet and indifferent to the injection); score 1 = mild pain (movement of tail and turning of head towards injection site); score 2 = moderate pain (light grunts and attempts to remove needle); and score 3 = severe pain (strong vocalization and attempts to escape, requiring vigorous physical restraint). Time from end of injection to lateral recumbency was recorded. The lateral auricular vein was catheterized as soon as the animals achieved lateral recumbency. Anesthesia was induced (8 mg/kg) and maintained using IV thiopental sodium (Pentothal sodium 1 gr^®^; Intervet, Aprilia, LT, Italy) based on assessment of anesthetic depth using the following criteria: eye position, degree of palpebral reflex, heart rate, respiratory rate and spontaneous movement. Immediately after the induction of anesthesia, a pain relief was administered (IM ketoprofen; 3 mg/kg; Vet-Ketofen 10%^®^, Ceva salute Animale S.p.A., Agrate Brianza Italia), a marker was used to draw an ID number for the identification of each animal. The eutopic testis and the inguinal incision site were infiltrated with lidocaine 2% (Lidocaina 2%^®^, ESTEVE S.p.A., Milano, Italia) five minutes before castration. The pigs were submitted to surgery to remove the retained testicle and the normally descended testes. The same experienced veterinarian performed all the surgeries. A closed castration technique was used and a laparotomy over the inguinal site was performed in order to remove the intra-abdominal retained testis. A simple interrupted suturing technique using an absorbable suture was used to close the surgical wounds. Rectal temperature was measured before and after surgery.

### 2.3. Video Recordings

Pigs were filmed the day before the procedure (pre-surgery) and 6 h after surgery (6 h post-surgery). Two high Definition Cameras (Panasonic, HDC-SD99, Panasonic, Japan), placed outside the housing pen at opposite sides, were used to record both behavior and face of the pigs without interfering with their normal behavior. At each time point (pre- and 6 h post-surgery), the video recording session lasted one hour. All the recordings were carried out in the afternoon between 2 p.m. and 6 p.m.

#### 2.3.1. Behavior Recording

Video sequences were recorded using a High Definition Camera with a 28 mm wide angle objective lens (Panasonic, HDC-SD99, Panasonic, Japan), the day before surgery in the evening (baseline observation, pre-surgery) and 6 h following the procedure (6 h post-surgery). The camera was located outside the pen to limit the disturbance of animals and to avoid possible modifications of their normal behavior.

A scan sampling method with instantaneous sampling was used to record behavioral states [14,15]. Each video lasted one hour; a 60 s sample interval was used for the following behavioral states:

- activity: eating, drinking, manipulating objects, walking;
- inactivity: lying position (sternal/lateral) and contact with other pigs (no contact/<50% body/>50% body).

Consequently, a total of 60 scans for each video (pre- and 6 h post-surgery) was obtained. The aim of the sampling procedure was to obtain an estimate of the proportion (or percentage) of individuals in each behavioral category pre- and 6 h post-surgery.

#### 2.3.2. Piglet Grimace Scale (PGS) Recording and Scoring

**Figure 1:**
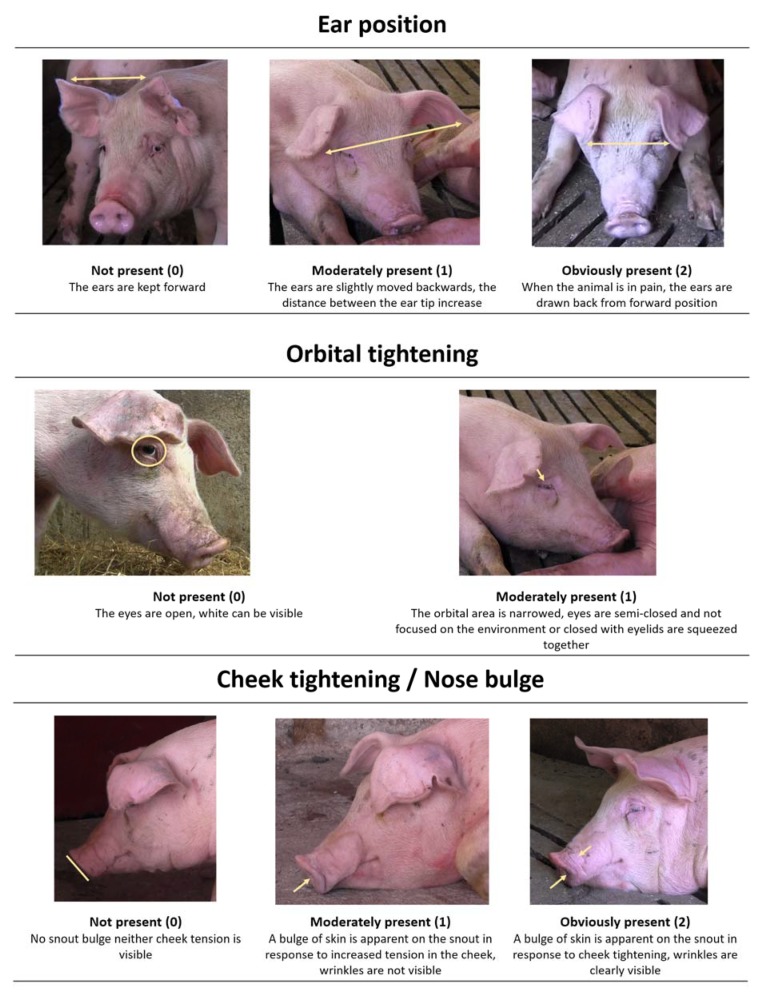
The handout distributed to Piglet Grimace Scale (PGS) assessors with images and explanations for each of the 3 Facial Action Units (FAUs). Ear position and check tightening/nose bulge is scored according to whether it is not present (0), moderately present (1), and obliviously present (2). While orbital tightening is scores on a 2-point scale: Not present (0), present (1).

Still frames of the face of each pig were extracted from the videos on every occasion they directly faced the video camera. A non-participating treatment- and time-point-blind assistant with no experience of assessing pain in pigs randomly selected 60 still images (image set). In order to maintain a balanced design for the statistical analysis, the image set comprised three images of each pig at each time point (pre- and 6 h post-surgery), for a total of six images per pig. Images were organized in a random order using the software Random.org (https://www.random.org/sequences/). A detailed hand out with the description of each FAU and the scoring system (Figure 1) was prepared and then distributed to three condition- and time-point-blind assessors to be used as training material for picture scoring. Assessors, who received a common training, had the same professional background and experience (veterinarian or graduated in animal production, with at least three years of experience in intensive pig farming). For each picture, the assessor was asked to give a score for each FAU. The PGS score was determined by adding the individual scores for each of the three action units; therefore, the maximum pain score using the PGS was 5.

### 2.4. Statistical Analysis

Statistical analysis was performed using SPSS 25 (SPSS Inc., Chicago, IL, USA). Statistical significance was accepted at *p* ≤ 0.05. The reliability of the PGS was determined using the inter-class correlation coefficient (ICC) to compare each FAU score and the PGS score across the two assessors. The following guidelines were used to interpret the ICC measures [16]: less than 0.40: poor; between 0.40 and 0.59: fair; between 0.60 and 0.74: good; between 0.75 and 1.00: excellent. The data were tested for normality and homogeneity of variance using the Kolmogorov–Smirnov and Levene test, respectively. The Paired Sample *t*-test was used to determine whether the PGS score changed between pre- and 6 h post-surgery. For behavioral recordings, the percentage of individuals per scan for each behavioral category pre- and 6 h post-surgery was calculated. The Wilcoxon test was used to investigate differences in behavior shown pre- and 6 h post-surgery.

## 3. Results

The injection of a sedative solution was not painful as no pain on drug injection (score 0) was shown by pigs. The combination of azaperone and ketamine rapidly induced a deep sedation with lateral recumbency within 3 min. No animals vomited following sedation, and anesthesia induction with sodium thiopental was rapid and smooth. Only one pig showed transient apnea. Surgical procedure was completed in all animals without surgical or anesthetic complications and all pigs survived. No animal required rescue analgesia post-operatively. The average time required to perform the surgery was 18.3 ± 2.4 (mean ± SD) minutes. Time from the end of the surgery to sternal recumbency was 35.3 ± 6.1 (mean ± SD) minutes, and time from sternal recumbency to standing was 10.4 ± 6.7 (mean ± SD) minutes.

**Figure 2:**
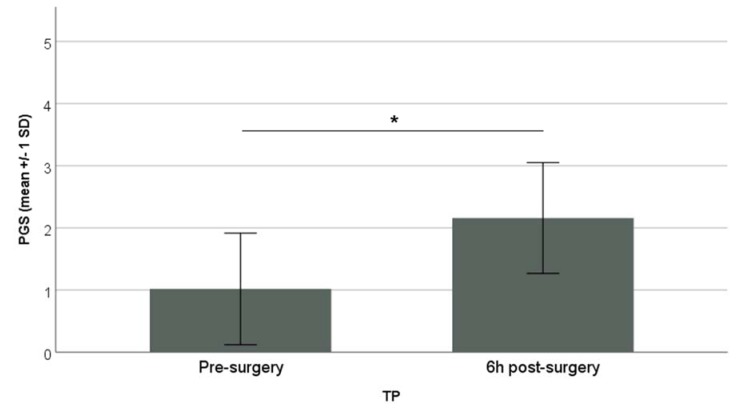
The PGS score is presented on the y-axis (mean ± 1 SD) with the pre- and 6 h post-surgery recordings on the x-axis (Paired sample *t*-test; * *p* = 0.02).

The PGS demonstrated an excellent inter-observer reliability with an overall Interclass Correlation Coefficient (ICC) value of 0.87. The individual FAUs showed excellent reliability with ICC values of: 0.73 for ear position, 0.90 for orbital tightening, 0.81 for cheek tightening/nose bulge. The PGS score pre-surgery was 1.02 ± 0.90 (mean ± SD) and increased significantly to 2.16 ± 0.89 (mean ± SD) 6 h post-surgery (Paired sample *t*-test, *p* = 0.02) (Figure 2).

**Figure 3:**
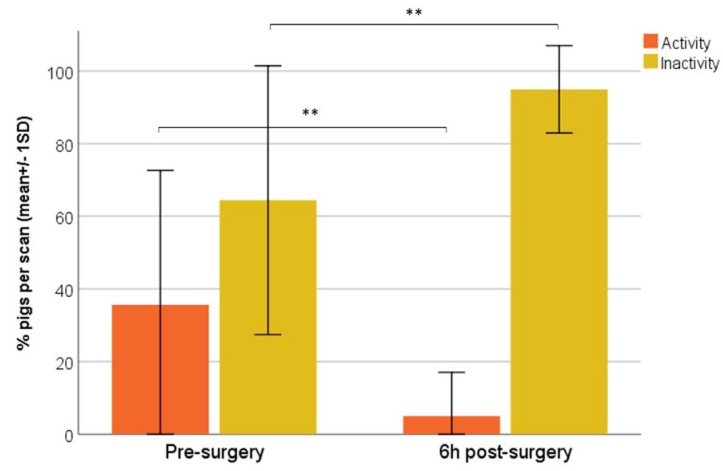
Mean scan percentages (±1 SD) of activity and inactivity of pigs pre- and 6 h post-surgery. (Wilcoxon test; ** *p* = 0.000).

Compared to pre-surgery, the percentage of activity significantly decreased, while inactivity significantly increased 6 h post-castration (Wilcoxon, *p* = 0.000) (Figure 3).

Pigs 6 h after surgery were lying significantly more in sternal position than pre-surgery, 74% and 38% respectively (Wilcoxon, *p* = 0.000). Before surgery, pigs preferred to lie down not in contact with other pigs, while 6 h post-surgery pigs settled in contact with pen mates (Wilcoxon, *p* = 0.000).

## 4. Discussion

According to the legislation in force in the European Union (Council Directive 2008/120/EC of 18 December 2008), pigs after their seventh day of life should be castrated under general anesthesia and should receive additional prolonged analgesia, e.g., ketoprofen. Viscardi and Turner demonstrated that ketoprofen was ineffective in alleviating pain resulting from surgical castration in 5-days piglets [17]. In our study, the anesthesia protocol included ketamine and pre-incisional and intratesticular and lidocaine. Ketamine is a non-competitive NMDA receptor antagonist, conferring it its specific properties (amnesic and psychosensory effects, analgesia, and neuroprotection). Lidocaine is a local analgesic that blocks the voltage-gated sodium channels, leading to a reversible block of action potential propagation. Both of these drugs allowed for a good management of intra and postoperative pain.

Physical restraint is difficult in pigs and a source of stress. Furthermore, the placement of an intravenous catheter is challenging if the animal is not properly sedated [18,19]. Therefore, sedatives are generally administered intramuscularly in pigs [20].

In the present study, the anesthetic and analgesic protocol included medicines selected from those reported in the Commission Regulation (EU) No 37/2010 of 22 December, 2009, as they can be used in animals intended for meat production. Several anesthetic drugs are still not approved by Italian legislation, including buprenorphine. Therefore, we could not include a blinded treatment group that received this opioid, even if it has been reported to be highly effective in alleviating castration-associated pain behaviors and facial grimacing in piglets [12,21]. For both ethical and welfare reasons, a control group undergoing general anesthesia without surgery was not included in this study. Thus, it proved impossible to obtain pain-free post-sedation pictures to exclude that the general anesthesia could affect the PGS score. Furthermore, as data were collected on-farm, no experimental animals undergoing general anesthesia without surgery could be included. In piglets, the sedative effects of injected general anesthetics can last from 2 to 50 min [4,18,22,23,24,25], therefore a 6 h post-surgery interval was selected to monitor pain as the sedative effect would be ended. A further validation of the PGS in growing pigs could be helpful to clarify the effect of general anesthesia on pig facial expression, taking into account whether some FAUs can be more affected than others.

The findings of this study suggest that the Piglet Grimace Scale (PGS) is a potentially effective method to assess pain in growing pigs undergoing on-farm cryptorchidectomy. Assessors blinded to time and condition attributed significantly higher PGS scores to pigs 6 h post-surgery. This was coherent with the observation that 6 h post-surgery pigs were less active and preferred to lie down in contact with pen mates.

Similar to results reported for other grimace scales [26,27,28,29], the inter-observer reliability of the PGS score in the present study was excellent, confirming that the use of a manual showing each FAU score as training material of assessors is sufficient to reliably apply this method. As humans instinctively tend to focus on the face when assessing pain in people and other animals [30,31], the PGS could represent a promising tool to identify on-farm, event-related pain. In laboratory animal species, as well as veterinary clinics, grimace scales are already being used through direct observation to assess pain [32,33,34].

As the PGS was originally developed for piglets, in the present study some changes were proposed to ensure that this tool could be applicable also to growing pigs. Thus far, some aspects need review. There is a lack of repeatability in stress/fear/pain studies in domestic animals, including pigs [35], in which case the absence of a clear pain assessment tool applicable across pigs of different age and weight arguably factors in. Existing literature reported results from experiments on 5-day-old piglets [9,11,12], minipigs [36], or straight-eared cross-breeds [37]. The original PGS describes the FAU “ear position” as “the ears are drawn back from forward (baseline) position” [12]; however, our observations failed to record unequivocal backward ear movements, which could be explained by the conformation of our older pigs. Early after birth, piglets offer a relatively wide range of facial expressions [12], based on their precocial development allowing immediate coordinated muscle movement, a relative lack of adipose tissue, and the thinness of their skin compared to their older counterparts [38,39]. It is especially true in high-gain breeds and cross-breeds, that rapid growth then changes the morphology of the face, some breeds seeing their ear pinnae fold under their own weight [40]. While these breeds tend to have a faster muscle growth and higher muscle cellularity [41], muscles pertaining to the scutiform cartilage in pigs, such as the parotid-auricular muscle, could in time become less prominent and reduce the possibility of ear movement, especially since swine dermis and epidermis get exceptionally thick [42].

Regarding the pigs’ behavior, we found that surgery induced alterations in pain-related behaviors. Six hours after surgery, castrated pigs were more often lying down, sitting or standing inactive compared to pre-surgery. Furthermore, when lying down, pigs chose to be more in contact with pen mates. Inactivity and social cohesion have been observed in piglets—as well as in other animals—undergoing painful husbandry procedures [43,44]. These behaviors can be interpreted as protective, allowing animals to avoid or reduce the stimulus from painful tissues [43,44]. The increase in lying in sternal position compared to lateral position observed after surgery may be interpreted in the same way. Another possible reason for lying in contact with conspecifics could be thermoregulation; it has been reported that lying behavior is an important thermoregulatory mechanism in pigs. When the temperature lowers, pigs adapt their behavior by huddling and lying down [45]. Since a grimace scale has never been applied to growing pigs, the analysis is essential to understand whether the animal is in pain.

## 5. Conclusions

This study presents the first attempt to apply the grimace scale to growing pigs undergoing on-farm surgery. The PGS was determined to be a potentially effective method to assess pain associated with cryptorchidectomy. The recognition and alleviation of pain is an emerging welfare issue in pig production, concerning both acute pain induced by management procedures, and chronic pain associated with conditions such as gastric ulcers or lameness. Limited information is available on behavioral and physiological measures of pain in pigs and research has been lately focusing on the inclusion of affective components in farm animal pain assessment. A standardized pain scale based on facial expressions may be a practical quantitative tool for pain evaluation in the field. However, further validation studies are required to investigate the use of PGS in growing pigs, and should examine other potentially painful procedures, effective pharmacological treatments, and its application by farmers as a pen-side tool for pain identification.
